# Combined Chaetocin/Trichostatin A Treatment Improves the Epigenetic Modification and Developmental Competence of Porcine Somatic Cell Nuclear Transfer Embryos

**DOI:** 10.3389/fcell.2021.709574

**Published:** 2021-10-06

**Authors:** Pil-Soo Jeong, Hae-Jun Yang, Soo-Hyun Park, Min Ah Gwon, Ye Eun Joo, Min Ju Kim, Hyo-Gu Kang, Sanghoon Lee, Young-Ho Park, Bong-Seok Song, Sun-Uk Kim, Deog-Bon Koo, Bo-Woong Sim

**Affiliations:** ^1^Futuristic Animal Resource and Research Center (FARRC), Korea Research Institute of Bioscience and Biotechnology (KRIBB), Cheongju, South Korea; ^2^Department of Biotechnology, College of Engineering, Daegu University, Gyeongsan, South Korea; ^3^Department of Animal Science, College of Natural Resources and Life Science, Pusan National University, Miryang, South Korea; ^4^Department of Animal Science and Biotechnology, College of Agriculture and Life Science, Chungnam National University, Daejeon, South Korea; ^5^Department of Functional Genomics, University of Science and Technology, Daejeon, South Korea

**Keywords:** chaetocin, trichostatin A (TSA), somatic cell nuclear transfer (SCNT), porcine embryonic development, epigenetic reprogramming, zygotic genome activation, genomic imprinting

## Abstract

Developmental defects in somatic cell nuclear transfer (SCNT) embryos are principally attributable to incomplete epigenetic reprogramming. Small-molecule inhibitors such as histone methyltransferase inhibitors (HMTi) and histone deacetylase inhibitors (HDACi) have been used to improve reprogramming efficiency of SCNT embryos. However, their possible synergistic effect on epigenetic reprogramming has not been studied. In this study, we explored whether combined treatment with an HMTi (chaetocin) and an HDACi (trichostatin A; TSA) synergistically enhanced epigenetic reprogramming and the developmental competence of porcine SCNT embryos. Chaetocin, TSA, and the combination significantly increased the cleavage and blastocyst formation rate, hatching/hatched blastocyst rate, and cell numbers and survival rate compared to control embryos. In particular, the combined treatment improved the rate of development to blastocysts more so than chaetocin or TSA alone. TSA and combined chaetocin/TSA significantly reduced the H3K9me3 levels and increased the H3K9ac levels in SCNT embryos, although chaetocin alone significantly reduced only the H3K9me3 levels. Moreover, these inhibitors also decreased global DNA methylation in SCNT embryos. In addition, the expression of zygotic genome activation- and imprinting-related genes was increased by chaetocin or TSA, and more so by the combination, to levels similar to those of *in vitro*-fertilized embryos. These results suggest that combined chaetocin/TSA have synergistic effects on improving the developmental competences by regulating epigenetic reprogramming and correcting developmental potential-related gene expression in porcine SCNT embryos. Therefore, these strategies may contribute to the generation of transgenic pigs for biomedical research.

## Introduction

Somatic cell nuclear transfer (SCNT) is a powerful method that allows reprogramming of terminally differentiated cells to the totipotent state (Gurdon and Wilmut, 2011). SCNT has applications in many areas, including animal husbandry, biomedical research, and endangered animal conservation (Yang et al., 2007). Recently, the application of SCNT combined with genome editing technique has been attracting attention as an efficient method to produce transgenic animals (Hryhorowicz et al., 2020). In particular, pigs serve as excellent experimental models for biomedical research in areas such as human disease, bioreactors, and xenotransplantation due to anatomic, physiologic and genetic similarities between pigs and humans (Simon and Maibach, 2000; Prather et al., 2003; Schook et al., 2008; Giraldo et al., 2012). Although many transgenic pigs have been created, the cloning efficiency remains extremely low due to a low rate of full-term development, and high rates of pregnancy loss and fetal abnormalities (Wilmut et al., 2002; Whitworth and Prather, 2010; Chavatte-Palmer et al., 2012). Increasing evidence suggests that incomplete epigenetic reprogramming of the donor cell (in terms of DNA methylation and histone methylation/acetylation) causes low cloning efficiency (Dean et al., 2001; Matoba and Zhang, 2018; Simmet et al., 2020; Wang et al., 2020).

Small-molecule inhibitors have been used to improve epigenetic reprogramming and developmental competence of SCNT embryos. Several recent studies have found that histone methylation is involved in epigenetic reprogramming and the development of SCNT embryos. Pharmacological inhibition using histone methyltransferase inhibitors (HMTi) such as BIX-01294 (Huang et al., 2016), MM-102 (Zhang Z. et al., 2018), or RG108 (Zhai et al., 2018) significantly increases reprogramming and developmental efficiency of porcine SCNT embryos.

Chaetocin is a histone H3 lysine 9 trimethylation (H3K9me3)-specific methyltransferase inhibitor that inhibits the expression of the *SUV39H1* and *SUV39H2* genes (Greiner et al., 2005). H3K9me3 is associated with transcriptional repression and heterochromatin maintenance in mammalian cells (Wang et al., 2018). H3K9me3 levels are higher in SCNT embryos than in fertilized embryos, and aberrant expression of H3K9me3 in SCNT embryos is a major epigenetic barrier to epigenetic reprogramming and zygotic genome activation (ZGA), causing embryonic development failure in mice and humans (Matoba et al., 2014; Chung et al., 2015). We previously found aberrant levels of H3K9me3 in porcine SCNT embryos; chaetocin efficiently improved epigenetic reprogramming and developmental competence by inhibiting not only histone methylation but also DNA methylation (Jeong et al., 2020).

Histone deacetylase inhibitors (HDACi) such as trichostatin A (TSA) (Himaki et al., 2010), oxamflatin (Hou et al., 2014), and scriptaid (Zhao et al., 2009) have been used to regulate epigenetic modifications and improve developmental potential in SCNT embryos. TSA is commonly used to repress histone deacetylation, enhancing the transcriptional activity and full-term development of pig SCNT embryos (Li J. et al., 2008). However, although several studies have reported positive effects of HMTi or HDACi, any synergistic effects of such chemicals remain unknown. In the present study, we evaluated the effects of chaetocin, TSA, and their combination on developmental competence, H3K9me3 and histone H3 lysine 9 acetylation (H3K9ac), 5-methlycytosine (5-mc) levels, and ZGA- and imprinting-related gene expression in porcine SCNT embryos.

## Materials and Methods

### Chemicals

Unless otherwise stated, all chemicals and reagents were purchased from Sigma-Aldrich Chemical Co. (St. Louis, MO, United States).

### Oocyte Collection and *in vitro* Maturation

Porcine ovaries were obtained from prepubertal gilts (mixed breed of Landrace, Yorkshire, and Duroc) at a nearby slaughterhouse and transported to the laboratory within 2 h in 0.9% (w/v) saline containing 75 μg/mL potassium penicillin G and 50 μg/mL streptomycin sulfate at 38.5°C. Cumulus-oocyte complexes (COCs) were aspirated from 3 to 7 mm diameter follicles using an 18-gauge needle connected to a 10 mL disposable syringe. COCs were washed three times in Tyrode’s Albumin Lactate Pyruvate-HEPES medium and approximately 50 COCs were matured in 500 μL *in vitro* Maturation (IVM) medium in a four-well multi-dish (Nunc, Roskilde, Denmark) for 44 h at 38.5°C under 5% CO_2_ in air. The IVM medium consisted of tissue culture medium 199 supplemented with 10% porcine follicular fluid, 0.57 mM cysteine, 10 ng/mL β-mercaptoethanol, 10 ng/mL epidermal growth factor, 10 IU/mL pregnant mare serum gonadotropin, and 10 IU/mL human chorionic gonadotropin. After 22 h, COCs were further cultured in IVM medium without hormones for another 22 h. After IVM, cumulus cells were removed by repeated pipetting with a 0.1% hyaluronidase. Metaphase II oocytes with visible polar body, regular morphology, and homogenous cytoplasm were used for all experiments.

### *In vitro* Fertilization and *in vitro* Culture

*In vitro* Fertilization (IVF) was performed in a modified Tris-buffered medium (mTBM). Metaphase II oocytes were washed three times in mTBM containing 2.5 mM caffeine sodium benzoate and 1 mg/mL bovine serum albumin (BSA), and 10–15 oocytes were placed into a 48 μL droplet of IVF medium at 38.5°C in 5% CO_2_ in air. To prepare spermatozoa, freshly ejaculated semen from duroc boar (DARBY AI center, Jochiwon, South Korea) was washed three times with sperm washing medium [Dulbecco’s phosphate-buffered saline (DPBS; Gibco-BRL, Grand Island, NY, United States) supplemented with 1 mg/mL BSA, 100 μg/mL penicillin G, and 75 μg/mL streptomycin sulfate]. After washing, 2 mL sperm washing medium was added to the spermatozoa pellet followed by incubation for 15 min at 38.5°C in 5% CO_2_ in air. After incubation, the supernatant was washed with mTBM and resuspended with 1 mL mTBM. Then, a 2 μL amount of the diluted spermatozoa were added to 48 μL mTBM containing 10–15 oocytes to a final concentration of 1.5 × 10^5^ spermatozoa/mL. The oocytes were co-incubated with the spermatozoa for 6 h at 38.5°C under 5% CO_2_ in air. After 6 h, the oocytes were stripped by gentle pipetting and cultured in *in vitro* Culture (IVC) medium (PZM-3 medium containing 4 mg/mL BSA) at 38.5°C in 5% CO_2_ in air.

### Somatic Cell Nuclear Transfer

Metaphase II oocytes in DPBS supplemented with 4 mg/mL BSA, 75 μg/mL penicillin G, 50 μg/mL streptomycin sulfate, and 7.5 μg/mL cytochalasin B (CB) were cut using a sharp pipette and the chromosomes removed via squeezing method under an inverted microscope (DMI 3000B; Leica, Wetzlar, Germany) equipped with a micromanipulator (NT-88-V3; Nikon Narishige, Tokyo, Japan). Porcine kidney cells were used as donor cells for SCNT. The kidney was obtained surgically from a neonatal pig (mixed breed of Landrace, Yorkshire, and Duroc, 2 days old, male) (Jeong et al., 2017, 2020). A single donor cell was injected into the perivitelline space. A single cell-oocyte couplet was placed between two parallel electrodes (CUY 5100-100; Nepa Gene) and activated by a single direct current pulse (0.24 kV/cm for 50 μs) using an Electro Cell Fusion generator. The fusion medium was 280 mM mannitol containing 0.2 mM MgSO_4_^.^7H_2_O and 0.01% (w/v) polyvinyl alcohol (PVA); the couplets were incubated at 38.5°C in 5% CO_2_ in air. After 2 h, oocyte-cell couplets that were completely fused (as observed under an inverted microscope) were selected and activated in IVC medium supplemented with 5 mg/mL CB and 2 mM 6-dimethylaminopurine for 4 h at 38.5°C in 5% CO_2_ in air. Activated embryos were transferred to IVC medium at 38.5°C in 5% CO_2_ in air. The cleavage and blastocyst formation rates were determined at 48 and 144 h after culture, respectively.

### Chemical Treatment

To confirm the effects of chaetocin, TSA, or their combination treatment during porcine SCNT embryo development, activated embryos were cultured in IVC medium with 0.5 nM chaetocin (Jeong et al., 2020) and/or 50 nM TSA (Zhang et al., 2007) for 24 h after activation.

### Indirect Immunofluorescence Assay

The pronuclear, two-, and four-cell stage embryos were washed in DPBS supplemented with 0.1% (w/v) PVA (DPBS-PVA) for 10 min and permeabilized in DPBS with 0.5% (v/v) Triton X-100 for 1 h at room temperature. For the staining of global methylation, permeabilized pronuclear, two-, and four-cell stage embryos were additionally stored in 1M HCl for 30 min at 38.5°C. Then they were washed three times in DPBS-PVA and transferred to blocking medium (DPBS with 4 mg/mL BSA) for 1 h at room temperature. The pronuclear, two-, and four-cell stage embryos were incubated with primary antibodies against H3K9me3 (1:1,000, Abcam, Cambridge, MA, United States), H3K9ac (1:200, Cell Signaling Technology, Beverly, MA, United States), or 5-mc (1:200, Calbiochem, San Diego, CA, United States) overnight at 4°C. After washing three times with DPBS containing 0.05% (v/v) Tween 20 (PBST), the pronuclear, two-, and four-cell stage embryos were incubated with the secondary antibody (Alexa Fluor 488 goat anti-rabbit IgG) for 1 h at room temperature. After washing three times with PBST, they were mounted on clean glass slides, stained with 4′,6′-diamidino-2-phenylindole (DAPI), and observed under a fluorescence microscope (Olympus, Tokyo, Japan). Approximately 5–10 pronuclear, two-, and four-cell stage embryos were subjected to immunocytochemistry in each experiment.

For CDX2 staining, blastocysts were fixed in 4% (v/v) paraformaldehyde overnight at 4°C and washed three times in DPBS-PVA for 10 min each time. The blastocysts were permeabilized in DPBS with 0.5% (v/v) Triton X-100 for 1 h at room temperature, washed three times in DPBS-PVA, and stored in DPBS-PVA supplemented with 1 mg/mL BSA (DPBS-PVA-BSA) at 4°C overnight. The blastocysts were blocked with 10% normal goat serum for 45 min and then incubated overnight at 4°C with the primary antibody (mouse monoclonal anti-CDX2; undiluted; Biogenex Laboratories Inc., San Ramon, CA, United States). Subsequently, the blastocysts were washed three times in DPBS-PVA-BSA for 10 min each time and incubated for 1 h at room temperature with the conjugated secondary antibody (Alexa-Fluor-488-labeled goat anti-mouse IgG; 1:200 in DPBS-PVA-BSA). After washing three times in DPBS-PVA-BSA for 10 min each time, DNA was stained with 2 μg/mL DAPI. DAPI-labeled and/or CDX2-positive nuclei were observed under a fluorescence microscope (Olympus). The cell numbers within the blastocysts were counted by DAPI- labeled or CDX2- positive nuclei (CDX2 expressing cell is TE, and opposite is ICM). The number of ICM cells was counted as the total cell number minus the number of TE cells. Approximately five to ten blastocysts per group were subjected to immunocytochemistry in each experiment.

### Quantitative Real-Time Polymerase Chain Reaction

Poly(A) mRNAs were extracted from approximately 20 four-cell stage embryos or blastocysts using a Dynabeads mRNA Direct Micro Kit (Invitrogen) according to the manufacturer’s protocol. Reverse transcription was performed using the PrimeScript RT Reagent Kit with the gDNA Eraser (Takara Bio Inc., Shiga, Japan) according to the manufacturer’s protocol. The resulting cDNA served as a template for PCR amplification. The PCR conditions were 95°C for 30 s, 60°C for 30 s, and 72°C for 30 s; followed by extension at 72°C for 5 min. The Mx3000P QPCR system (Agilent, Santa Clara, CA, United States) and the SYBR Premix Ex Taq (Takara Bio Inc.) were employed. The primers used are listed in Supplementary Table 1.

### Terminal Deoxynucleotidyl Transferase-Mediated dUTP-Digoxygenin Nick End-Labeling Assay

The TUNEL assay was performed using an *in situ* cell death detection kit (Roche, Basel, Switzerland). Blastocysts were washed three times in DPBS-PVA and fixed in 4% (v/v) paraformaldehyde overnight at 4°C. The blastocysts were permeabilized in DPBS with 0.5% (v/v) Triton X-100 at room temperature for 1 h. Non-specific binding sites were blocked by incubation for 1 h with DPBS containing 10 mg/mL BSA. The blastocysts were washed three times with DPBS-PVA and stained with fluorescein-conjugated dUTP and terminal deoxynucleotidyl transferase for 1 h at 38.5°C. The blastocysts were washed three times with DPBS-PVA, mounted on clean glass slides, and stained with DAPI. DAPI-labeled and/or TUNEL-positive nuclei were observed under a fluorescence microscope (Olympus). Total and apoptotic cell numbers per blastocyst were derived by counting the nuclei yielding blue (DAPI) and green (TUNEL) signals. Approximately five to ten blastocysts per treatment group were subjected to the TUNEL assays in each experiment.

### Statistical Analyses

All experiments were repeated at least three times. Data are expressed as means ± standard errors of the means (SEMs). Data were compared using ANOVA, followed by the Duncan multiple range test of Sigmastat software (SPSS Inc., Chicago, IL, United States). *P*-values < 0.05 were considered to indicate statistical significance.

## Results

### Chaetocin, Trichostatin A, and the Combination Improved the Developmental Competence of Porcine Somatic Cell Nuclear Transfer Embryos

**FIGURE 1:**
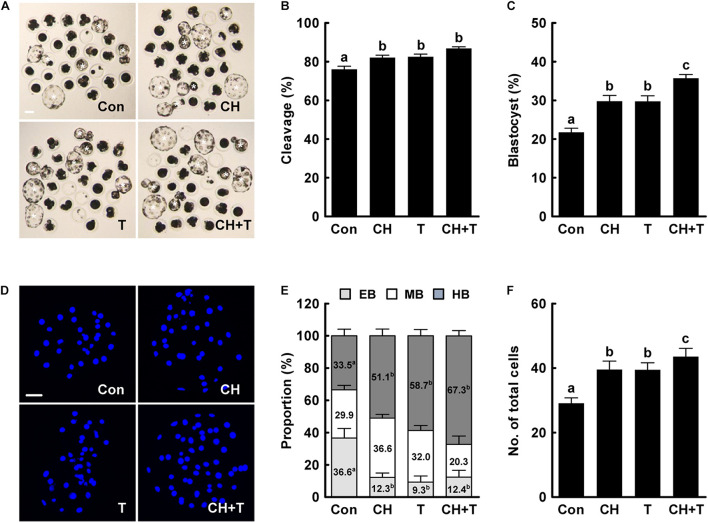
Effects of chaetocin, TSA, and the combination on *in vitro* development of porcine SCNT embryos. **(A)** Representative images of cultured blastocysts (white asterisks). Bar = 100 μm. Quantification of **(B)** the cleavage rate and **(C)** the blastocyst formation rate (*n* = 139 per group). **(D)** Representative nuclear-stained images of blastocysts. Bar = 50 μm. Quantification of **(E)** the various blastocyst stages and **(F)** total cell numbers (Con; *n* = 30, CH; *n* = 41, T; *n* = 41, CH + T; *n* = 49). The data are from four independent experiments and are means ± SEM. Values with different superscripts differ significantly (*P* < 0.05). Con, control; CH, chaetocin; T, TSA; EB, early blastocyst; MB, middle blastocyst; HB, hatching or hatched blastocyst.

**FIGURE 2:**
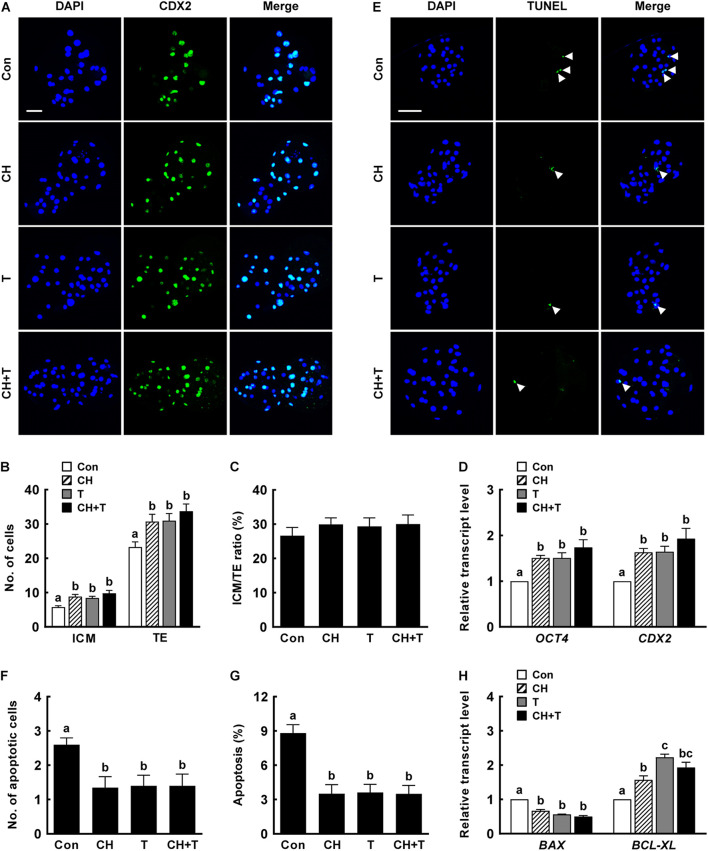
Effects of chaetocin, TSA, and the combination on the developmental competence of porcine SCNT embryos. **(A)** Representative immunofluorescent images of CDX2/DAPI-stained blastocysts. Green and blue fluorescence indicate CDX2 and nuclei, respectively. Bar = 50 μm. Quantification of **(B)** ICM and TE cell numbers and **(C)** ICM/TE ratios (*n* = 20 per group). **(D)** qRT-PCR data on embryonic development-related gene expression in blastocysts (*n* = 3 per group). **(E)** Representative TUNEL assay images of blastocysts. Green and blue fluorescence indicate the TUNEL label (white arrow) and nuclei, respectively. Bar = 50 μm. Quantification of **(F)** the apoptotic cell number and **(G)** the apoptosis rate (*n* = 20 per group). **(H)** qRT-PCR data on apoptosis-related gene expression in blastocysts (*n* = 3 per group). The data are from three independent experiments and are means ± SEM. Values with different superscripts differ significantly (*P* < 0.05). Con, control; CH, chaetocin; T, TSA; ICM; inner cell mass; TE, trophectoderm cell.

Previous studies have demonstrated that both treatment with 0.5 nM chaetocin and 50 nM TSA for 24 h after activation improves the epigenetic reprogramming and developmental competence of porcine SCNT embryos (Zhang et al., 2007; Cao et al., 2017; Jeong et al., 2020). Based on previous studies, we added 0.5 nM chaetocin and/or 50 nM TSA for 24 h after activation and allowed culture to continue for 6 days to assess the effects of chaetocin, TSA, and the combination on the developmental competence of porcine SCNT embryos. We measured cleavage and blastocyst formation rates, blastocyst hatching rates, and the total cell number of porcine SCNT embryos. All parameters were significantly increased by chaetocin and/or TSA treatment compared to control (Figure 1 and Supplementary Tables 2,3). In particular, combined chaetocin/TSA remarkably increased the blastocyst formation rate compared to either inhibitor alone (Figure 1C and Supplementary Table 2); the combination synergistically enhanced the developmental rate of porcine SCNT embryos. We also investigated blastocyst quality. CDX2 staining showed that chaetocin and/or TSA significantly increased the inner cell mass (ICM) and trophectoderm (TE) cell numbers of SCNT blastocysts compared to control, but did not affect the ICM:TE ratio (Figures 2A–C and Supplementary Table 4). Consistent with these results, chaetocin and/or TSA increased the expression levels of the *OCT4* and *CDX2* genes compared to control (Figure 2D). The TUNEL assay showed that chaetocin and/or TSA significantly decreased the apoptotic cell number and apoptosis rate of SCNT blastocysts compared to control (Figures 2E–G and Supplementary Table 5), as confirmed by Quantitative Real-Time Polymerase Chain Reaction (qRT-PCR) showing that chaetocin and/or TSA increased the expression level of an anti-apoptosis gene (*BCL-XL*) and reduced the expression level of a pro-apoptosis gene (*BAX*) compared to control (Figure 2H). These results suggest that chaetocin and TSA have synergistic effect on developmental competence of porcine SCNT embryo.

### Chaetocin, Trichostatin A, and the Combination Regulated H3K9me3 and H3K9ac Levels in Porcine Somatic Cell Nuclear Transfer Embryos

**FIGURE 3:**
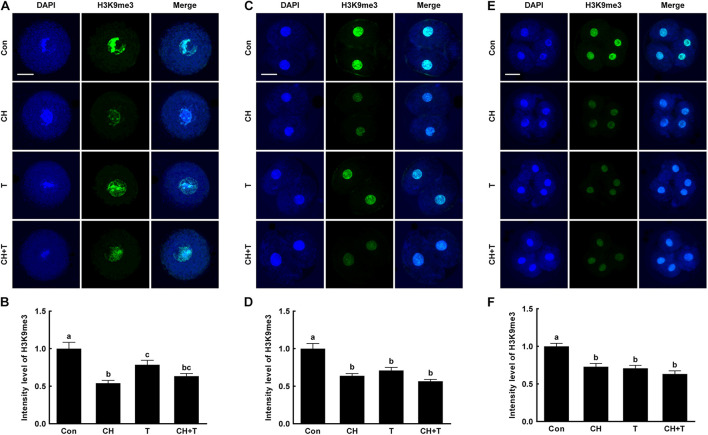
Effects of chaetocin, TSA, and the combination on H3K9me3 levels in porcine SCNT embryos. **(A)** Representative immunofluorescent images of H3K9me3 at the pronuclear stage. Green and blue fluorescence indicate H3K9me3 and nuclei, respectively. Bar = 50 μm. **(B)** Quantification of fluorescence intensity at the pronuclear stage (*n* = 20 per group). **(C)** Representative immunofluorescent images of H3K9me3 at the two-cell stage. Green and blue fluorescence indicate H3K9me3 and nuclei, respectively. Bar = 50 μm. **(D)** Quantification of fluorescence intensity at the two-cell stage (*n* = 17 per group). **(E)** Representative immunofluorescent images of H3K9me3 at the four-cell stage. Green and blue fluorescence indicate H3K9me3 and nuclei, respectively. Bar = 50 μm. **(F)** Quantification of fluorescence intensity at the four-cell stage (*n* = 20 per group). The data are from three independent experiments and are means ± SEM. Values with different superscripts differ significantly (*P* < 0.05). Con, control; CH, chaetocin; T, TSA.

**FIGURE 4:**
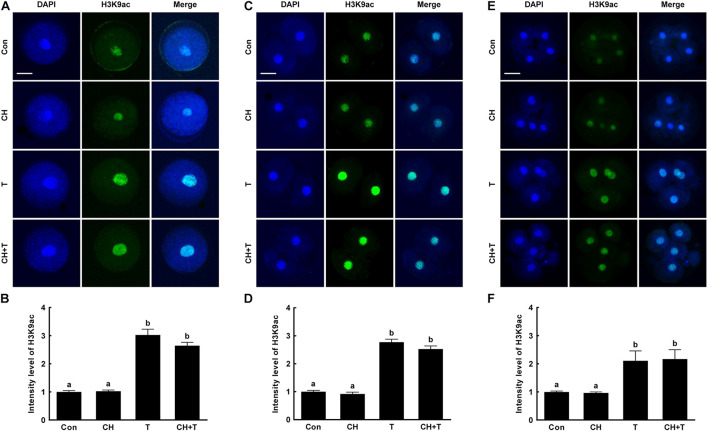
Effects of chaetocin, TSA, and the combination on H3K9ac levels in porcine SCNT embryos. **(A)** Representative immunofluorescent images of H3K9ac at the pronuclear stage. Green and blue fluorescence indicate H3K9ac and nuclei, respectively. Bar = 50 μm. **(B)** Quantification of fluorescence intensity at the pronuclear stage (*n* = 20 per group). **(C)** Representative immunofluorescent images of H3K9ac at the two-cell stage. Green and blue fluorescence indicate H3K9ac and nuclei, respectively. Bar = 50 μm. **(D)** Quantification of fluorescence intensity at the two-cell stage (*n* = 20 per group). **(E)** Representative immunofluorescent images of H3K9ac at the four-cell stage. Green and blue fluorescence indicate H3K9ac and nuclei, respectively. Bar = 50 μm. **(F)** Quantification of fluorescence intensity at the four-cell stage (*n* = 18 per group). The data are from three independent experiments and are means ± SEM. Values with different superscripts differ significantly (*P* < 0.05). Con, control; CH, chaetocin; T, TSA.

To investigate whether epigenetic modification improved the developmental competence of porcine SCNT embryos treated with chaetocin, TSA, or the combination, we measured H3K9me3 and H3K9ac levels by immunofluorescence (Figures 3, 4). Chaetocin (particularly) and/or TSA significantly reduced H3K9me3 level compared to control at the pronuclear stage. TSA and the combination treatment significantly increased the H3K9ac level compared to control and chaetocin treatment at the pronuclear stage. Moreover, these inhibitors also regulated H3K9me3 and H3K9ac levels similar to pronuclear stage at the two- and four-cell stage. Thus, the chaetocin/TSA combination had beneficial effects in terms of both H3K9me3 and H3K9ac modifications in porcine SCNT embryos.

### Chaetocin, Trichostatin A, and the Combination Regulated Global DNA Methylation in Porcine Somatic Cell Nuclear Transfer Embryos

**FIGURE 5:**
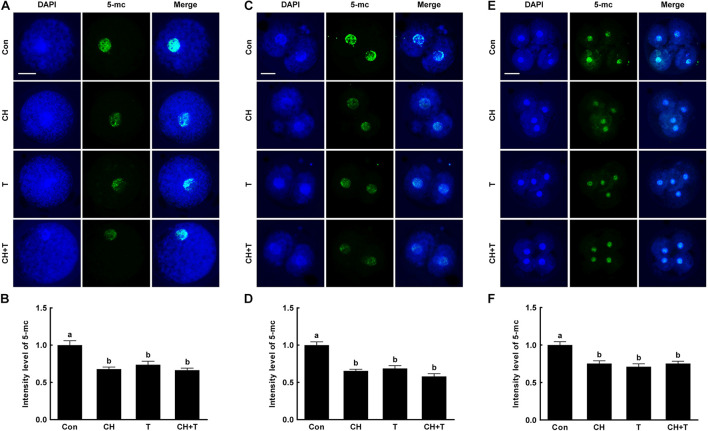
Effects of chaetocin, TSA, and the combination on global DNA methylation in porcine SCNT embryos. **(A)** Representative immunofluorescent images of 5-methylcytosine (5-mc) at the pronuclear stage. Green and blue fluorescence indicate 5-mc and nuclei, respectively. Bar = 50 μm. **(B)** Quantification of fluorescence intensity at the pronuclear stage (*n* = 20 per group). **(C)** Representative immunofluorescent images of 5-mc at the two-cell stage. Green and blue fluorescence indicate 5-mc and nuclei, respectively. Bar = 50 μm. **(D)** Quantification of fluorescence intensity at the two-cell stage (*n* = 20 per group). **(E)** Representative immunofluorescent images of 5-mc at the four-cell stage. Green and blue fluorescence indicate 5-mc and nuclei, respectively. Bar = 50 μm. **(F)** Quantification of fluorescence intensity at the four-cell stage (*n* = 18 per group). The data are from three independent experiments and are means ± SEM. Values with different superscripts differ significantly (*P* < 0.05). Con, control; CH, chaetocin; T, TSA.

A previous study reported that Abnormal DNA methylation is a major cause of developmental defects in SCNT embryos (Peat and Reik, 2012). To examine whether chaetocin and TSA affect global DNA methylation in porcine SCNT embryos, we measured 5-mc levels, an indicator of DNA methylation, by immunofluorescence (Figure 5). Chaetocin and/or TSA significantly reduced 5-mc level compared to control at the pronuclear, two-, and four-cell stage. These results indicated that chaetocin and TSA improve epigenetic reprogramming by regulating global DNA methylation in porcine SCNT embryos.

### Chaetocin, Trichostatin A, and the Combination Affected the Expression of Zygotic Genome Activation-Related Genes in Porcine Somatic Cell Nuclear Transfer Embryos

**FIGURE 6:**
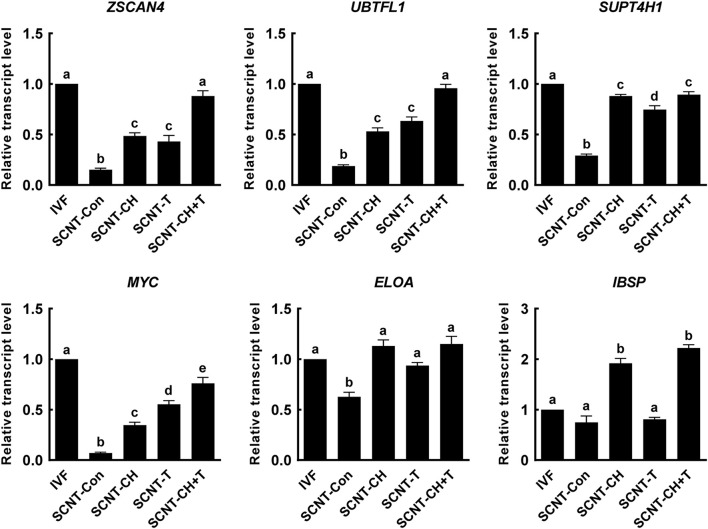
Effects of chaetocin, TSA, and the combination on the ZGA in porcine SCNT embryos. The data are from three independent experiments and are means ± SEM. Values with different superscripts differ significantly (*P* < 0.05). IVF, *in vitro* fertilization; SCNT, somatic cell nuclear transfer; Con, control; CH, chaetocin; T, TSA.

Aberrant histone methylation or acetylation interfere with normal ZGA induction in SCNT embryos (Matoba et al., 2014; Inoue et al., 2015). To explore whether chaetocin, TSA, or the combination regulated the ZGA of porcine SCNT embryos via epigenetic modification, we selected six ZGA-related genes that are known to be required for normal embryonic development (Matoba et al., 2014; Chung et al., 2015; Liu et al., 2018; Zhu et al., 2021) and investigated the expression levels of these genes at the four-cell stage using qRT-PCR (Figure 6). The expression levels of the *ZSCAN4*, *UBTFL1*, *SUPT4H1*, *MYC*, *ELOA*, and *IBSP* were markedly decreased in SCNT embryos compared to IVF embryos, but such impairments were significantly rescued by chaetocin and/or TSA. Notably, combination treatment afforded the highest expression of ZGA-related genes.

### Chaetocin, Trichostatin A, and the Combination Altered Expression of Imprinting-Related Genes in Porcine Somatic Cell Nuclear Transfer Embryos

**FIGURE 7:**
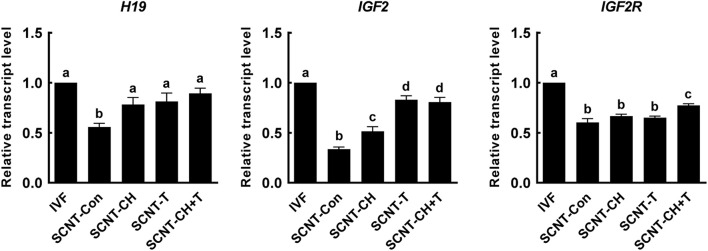
Effects of chaetocin, TSA, and the combination on the genomic imprinting in porcine SCNT embryos. The data are from three independent experiments and are means ± SEM. Values with different superscripts differ significantly (*P* < 0.05). IVF, *in vitro* fertilization; SCNT, somatic cell nuclear transfer; Con, control; CH, chaetocin; T, TSA.

Genomic imprinting is important in terms of successful embryonic development and fetal growth (Lee et al., 2006). To explore the effects of chaetocin, TSA, and the combination on genomic imprinting in porcine SCNT embryos, we investigated the expression levels of imprinting-related genes in blastocysts using qRT-PCR (Figure 7). The expression levels of the imprinting genes *H19*, *IGF2*, and *IGF2R* were significantly lower in SCNT than IVF embryos. However, the expression levels of *H19* and *IGF2* were significantly rescued by chaetocin, TSA, or the combination. In addition, the expression level of *IGF2R* was significantly increased by the inhibitor combination, but not by either inhibitor alone.

## Discussion

To date, many studies have reported that small-molecule inhibitors including HMTi and HDACi enhance the development of porcine SCNT embryos, but combined effect of these inhibitors has not been explored. In the present study, we found that the inhibitor combination synergistically enhanced epigenetic reprogramming and the developmental competence of porcine SCNT embryos. Moreover, the combination corrected the expression levels of ZGA- and imprinting-related genes.

Epigenetic reprogramming (DNA methylation and histone modification) affects gene transcription and chromatin structure during embryonic development (Matoba and Zhang, 2018). SCNT embryos exhibited developmental defects attributable to aberrant epigenetic reprogramming (abnormally high-level DNA and histone methylation, and low-level histone acetylation) compared to fertilized embryos, compromising SCNT embryo development (Czernik et al., 2019). H3K9me3 constitutes a major epigenetic barrier in mammalian SCNT embryos; microinjection of mRNA encoding the H3K9me3-specific demethylase of the KDM family significantly improved epigenetic reprogramming efficiency and SCNT embryo development (Matoba et al., 2014; Chung et al., 2015; Liu et al., 2018). Recently, chaetocin was reported to significantly increase epigenetic reprogramming and SCNT embryo development by reducing H3K9me3 levels in pigs (Jeong et al., 2020; Weng et al., 2020). Histone acetylation is also an important feature of epigenetic reprogramming. TSA has been widely used to upregulate histone acetylation and increase the developmental efficiency of SCNT embryos (Cervera et al., 2009; Akagi et al., 2014; Huan et al., 2015). Consistent with previous studies, we found that chaetocin, TSA, and the combination significantly increased cleavage and blastocyst formation rates, and the hatching blastocyst rate in porcine SCNT embryos. All treatments significantly increased blastocyst quality in terms of the ICM, TE, total cell numbers, expression levels of the *OCT4* and *CDX2* genes, and the apoptosis rate, compared to control. Notably, the combination treatment best enhanced almost all measures of developmental competence. These results indicate that combined chaetocin and TSA synergistically improved the developmental competence of porcine SCNT embryos.

Given that synergism was in play, we assumed that both inhibitors would affect histone methylation and acetylation. Unexpectedly, although TSA affected the levels of both H3K9ac and H3K9me3 (compared to control), chaetocin did not affect the H3K9ac level, but decreased the H3K9me3 level (only), consistent with a previous study (Zhang Y. M. et al., 2018). However, the combination of chaetocin and TSA significantly decreased the H3K9me3 level and increased the H3K9ac level, improving developmental competence.

Global DNA methylation is an important biological event associated with maintenance of genome stability, X-chromosome inactivation, and genomic imprinting in mammals (Sulewska et al., 2007). In particular, DNA methylation also plays a crucial role in epigenetic reprogramming, gene expression and normal embryonic development during early embryogenesis (Smith and Meissner, 2013). After fertilization, DNA methylation patterns are dynamically remodeled, characterized by the erasure of most methylation marks from the zygote to blastocyst stage followed by the establishment of the embryonic methylation pattern (Fulka et al., 2004; Smith and Meissner, 2013). However, SCNT embryos exhibited abnormal DNA methylation patterns with higher levels compared to fertilized embryos and subsequent low development efficiency. Thus, several inhibitors have been used to regulate DNA methylation in order to improve epigenetic reprogramming and developmental competence in SCNT embryos (Xu et al., 2013; Taweechaipaisankul et al., 2019; Wu et al., 2019). In this study, we show that chaetocin and TSA significantly reduced 5-mc levels, an indicator of DNA methylation, and subsequent increased embryonic development in porcine SCNT embryos, consistent with previous studies (Li X. et al., 2008; Cao et al., 2017; Jeong et al., 2020). These results suggest that chaetocin and TSA improves epigenetic reprogramming and developmental competence by regulating not only histone modification but also global DNA methylation during SCNT embryo development.

The major ZGA developmental transition is associated with degradation of maternal proteins and mRNAs, and initiation of mRNA synthesis in the newly formed zygotic genome. ZGA occurs at the two-cell stage in mice and at the four- to eight-cell stage in pigs, cattle, and humans (Hyttel et al., 2000; Schultz, 2002). However, SCNT embryos undergo abnormal gene expression and developmental arrest at the ZGA stage, which is reported to be due to aberrant epigenetic reprogramming (Loi et al., 2016). In particular, H3K9me3 associated with transcriptional repression is enriched in reprogramming resistant regions at the ZGA stage resulted in developmental arrest of SCNT embryos; injection of a KDM4 family enzyme eliminated abnormal gene expression and improved developmental competence, indicating that abnormally high H3K9me3 is major cause of ZGA failure in SCNT embryos (Matoba et al., 2014; Chung et al., 2015). Moreover, mRNA expression profile analysis demonstrated the abnormal expression of epigenetic modification enzymes such as DNA methylation and histone methylation/acetylation in SCNT embryos compared to *in vivo* embryos, suggesting that ZGA failure is related to incomplete reprogramming of SCNT embryos (Zhang Z. et al., 2018). Based on these previous studies, we confirmed that combined chaetocin and TSA treatment significantly reduced H3K9me3 and 5-mc levels and increased H3K9ac levels. Moreover, we examined the expression levels of ZGA-related genes (*ZSCAN4*, *UBTFL1*, *SUPT4H1*, *MYC*, *ELOA*, and *IBSP*) at the four-cell stage. The SCNT embryos exhibited significantly lower expression of such genes than IVF embryos, but expression was rescued by chaetocin, TSA, or the combination; in particular, the combination treatment induced the highest expression levels of ZGA related genes. Collectively, these results suggest that improvement of epigenetic reprogramming by combined chaetocin/TSA treatment rescued impaired embryonic genome activation in porcine SCNT embryos.

Genomic imprinting is important in terms of successful preimplantation embryonic development, fetal and placental growth (Reik and Walter, 2001). SCNT embryos were observed to have aberrant genomic imprinting (abnormal methylation) that induces dysregulation of gene expression and developmental abnormalities (Mann et al., 2003; Ogawa et al., 2003). The *H19*, *IGF2*, and *IGF2R* genes are imprinted in pigs (Shen et al., 2012). Previous study reported that TSA corrects aberrant expression of the *H19*/*IGF2* genes in porcine SCNT embryos (Huan et al., 2015), and combined treatment with a DNA methyltransferase inhibitor (RG108) and HDACi (Scriptaid) synergistically corrected abnormal imprinting (Xu et al., 2013). Consistent with previous studies, we found that the expression levels of *H19* and *IGF2* genes in SCNT blastocysts were significantly lower than in IVF embryos, but the levels were significantly rescued by chaetocin, TSA, or the combination. Notably, *IGF2R* expression was rescued by only the combined treatment. Therefore, we suggest that the inhibitors combination optimally corrects disrupted genomic imprinting in pigs.

Taken together, we found that combination of chaetocin and TSA improved developmental competence, appropriately regulated H3K9me3, H3K9ac, and global DNA methylation and rescued the expression of ZGA- and imprinting-related genes. These findings suggest that the combination of chaetocin and TSA may optimally regulate epigenetic reprogramming to enhance the developmental competence of porcine SCNT embryos, improving the production of transgenic pigs for biomedical research.

## Data Availability Statement

The original contributions presented in the study are included in the article/Supplementary Material, further inquiries can be directed to the corresponding author/s.

## Author Contributions

P-SJ designed the study, performed the experiments, analyzed the data, and wrote the manuscript. H-JY, S-HP, MG, YJ, MK, H-GK, and SL performed the experiments. Y-HP and B-SS performed the experiments and collected the data. S-UK acquired financial, analyzed the data, and discussed the study. D-BK and B-WS designed the study, supervised the study, discussed the study, and wrote the manuscript. All authors have read and agreed to the published final version of this manuscript.

## Conflict of Interest

The authors declare that the research was conducted in the absence of any commercial or financial relationships that could be construed as a potential conflict of interest.

## Publisher’s Note

All claims expressed in this article are solely those of the authors and do not necessarily represent those of their affiliated organizations, or those of the publisher, the editors and the reviewers. Any product that may be evaluated in this article, or claim that may be made by its manufacturer, is not guaranteed or endorsed by the publisher.
