# Supplementary material for: Combined Chaetocin/Trichostatin A Treatment Improves the Epigenetic Modification and Developmental Competence of Porcine Somatic Cell Nuclear Transfer Embryos
Source: Front Cell Dev Biol. 2021 Oct 6;9:709574. doi: 10.3389/fcell.2021.709574 (PMC8526721; doi:10.3389/fcell.2021.709574)
Supplement: Supplementary file 1 [file Data_Sheet_1.PDF]

Supplementary Table 1. Primer sequences for qRT-PCR

Gene	Primer sequences	GenBank accession no.	Product size (bp)
OCT4	F: 5’- AGT GAG AGG CAA CCT GGA GA -3’<br>R: 5’- ACT GCT TGA TCG TTT GCC CT -3’	NM_001113060.1	151
CDX2	F: 5’- GGC AGC CAA GTG AAA ACC AG -3’<br>R: 5’- GCC TTT CTC CGA ATG GTG AT -3’	NM_001278769.1	119
BAX	F: 5’- CGA TCT CGA AGG AAG TCC AG -3’<br>R: 5’- AAG CGC ATT GGA GAT GAA CT -3’	XM_003127290.5	251
BCL-XL	F: 5’- AGG GCA TTC AGT GAC CTG AC -3’<br>R: 5’- TGG ATC CAA GGC TCT AGG TG -3’	NM_214285.1	242
ZSCAN4	F: 5’- AAACGCAGCAAGGATGAGAT -3’<br>R: 5’- GCCACTTGCATTCCATTTCT -3’	XM_021097584.1	108
UBTFL1	F: 5’- GAGCAGCCATGAACAAGACA -3’<br>R: 5’- TCCCTCTCAGCTGGTGAGTT -3’	XM_021102676.1	158
SUPT4H1	F: 5’- AGTCGAGGAGTGGCCTACAA -3’<br>R: 5’- CTCCCTCAACAGCCTGAGTC -3’	XM_003131646.4	182
MYC	F: 5’- CTGCCAAGAGGGCTAAGTTG -3’<br>R: 5’- GTCCGCCTCTTGTCATTCTC -3’	NM_001005154.1	112
ELOA	F: 5’- ACCAGCCCTCATCAGATGTC -3’<br>R: 5’- GGAAGATCTGTGCTCCTTGC -3’	XM_003356197.4	195
IBSP	F: 5’- CGAGGGGGAGTATGAACAGA -3’<br>R: 5’- CCTCGTAGGCTCGGTAACTG -3’	XM_003129337.3	107
H19	F: 5’- CTCAAACGACAAGAGATGGT -3’<br>R: 5’- AGTGTAGTGGCTCCAGAATG -3’	AY044827	147
IGF2	F: 5’- GGCATCGTGGAAGAGTGCT -3’<br>R: 5’- CTGGGGAAGTTGTCCGGAAG -3’	NM_213883	128
IGF2R	F: 5’- CCTGGTTCTTTGTCCACGAT -3’<br>R: 5’- TACCCGATGGGTTCTTCTTG -3’	NM_001244473	117
GAPDH	F: 5’- CCC TGA GAC ACG ATG GTG AA -3’<br>R: 5’- GGA GGT CAA TGA AGG GGT CA -3’	NM_001206359.1	147

Supplementary Table 2. Effects of chaetocin, TSA, and the combination on the early development of porcine SCNT embryos

Groups	No. of embryos examined	Cleavage (%)	Blastocyst (%)	Total cell number
Control	139	106 (76.1±1.5)[a]	30 (21.8±1.1)[a]	31.4±1.7[a]
Chaetocin	139	114 (82.1±1.2)[b]	41 (29.8±1.5)[b]	37.5±2.1[b]
TSA	139	115 (82.6±1.3)[b]	41 (29.7±1.5)[b]	40.5±3.0[b]
Chaetocin+TSA	139	121 (87.0±0.8)[b]	49 (35.4±0.8)[c]	35.7±1.9[c]

Data are the mean ± SEM, and values with different superscript letter within a column differ significantly ($p < 0.05$).

Supplementary Table 3. Effects of chaetocin, TSA, and the combination on the post-blastulation development of porcine SCNT blastocysts

Groups	No. of blastocysts examined	Proportion of blastocysts developed to the following stages (%)		
		Early	Middle	Hatching/Hatched
Control	30	36.6±5.9[a]	29.9±2.7[a,c]	33.5±4.1[a]
Chaetocin	41	12.3±2.6[b]	36.6±2.4[a,b]	51.1±4.2[b]
TSA	41	9.3±3.7[b]	32.0±3.0[a,c]	58.7±3.9[b]
Chaetocin+TSA	49	12.4±4.2[b]	20.3±5.2[c]	67.3±3.3[b]

Data are the mean ± SEM, and values with different superscript letter within a column differ significantly ($p < 0.05$).

.

Supplementary Table 4. Effects of chaetocin, TSA, and the combination on the ICM/TE ratio in porcine SCNT blastocysts

Groups	No. of blastocysts examined	No. of nuclei			ICM/TE (%)
		ICM	TE	Total	
Con	20	5.8±0.4[a]	23.3±1.5[a]	29.1±1.7[a]	26.6±2.4
Chaetocin	20	8.9±0.6[b]	30.7±2.2[b]	39.6±2.6[b]	29.9±1.9
TSA	20	8.5±0.5[b]	31.0±2.1[b]	39.5±2.2[b]	29.4±2.5
Chaetocin+TSA	20	9.8±0.9[b]	33.8±2.1[b]	43.6±2.5[b]	30.0±2.6

Data are the mean ± SEM, and values with different superscript letter within a column differ significantly ($p < 0.05$).

Supplementary Table 5. Effects of chaetocin, TSA, and the combination on cell survival in porcine SCNT blastocysts

Groups	No. of blastocysts examined	No. of TUNEL-positive cells	Apoptosis (%)
Con	20	2.6±0.2[a]	8.8±0.7[a]
Chaetocin	20	1.4±0.3[b]	3.5±0.8[b]
TSA	20	1.4±0.3[b]	3.6±0.7[b]
Chaetocin+TSA	20	1.4±0.3[b]	3.5±0.7[b]

Data are the mean ± SEM, and values with different superscript letter within a column differ significantly ($p < 0.05$).
